# Association Between Gout and the Risk of Dementia: A Meta‐Analysis of Observational Studies and Biological Mechanisms

**DOI:** 10.1111/1756-185x.70582

**Published:** 2026-03-04

**Authors:** Yao‐Chin Wang, Abel Po‐Hao Huang, Chih‐Wei Huang, Md. Mohaimenul Islam, Woon‐Man Kung

**Affiliations:** ^1^ Department of Emergency Medicine Taipei Medical University Hospital Taipei Taiwan; ^2^ Graduate Institute of Injury Prevention and Control, College of Public Health Taipei Medical University Taipei Taiwan; ^3^ International Center for Health Information Technology, College of Medical Science and Technology Taipei Medical University Taipei Taiwan; ^4^ Division of Neurosurgery, Department of Surgery, College of Medicine, National Taiwan University Hospital National Taiwan University Taipei Taiwan; ^5^ Graduate Institute of Biomedical Informatics, College of Medical Science and Technology Taipei Medical University Taipei Taiwan; ^6^ Clinical Big Data Research Center, Taipei Medical University Hospital Taipei Medical University Taipei Taiwan; ^7^ Division of Outcomes and Practice Advancement, Department of Pharmacy Practice, School of Pharmacy and Pharmaceutical Sciences University at Buffalo Buffalo New York USA; ^8^ Division of Neurosurgery, Department of Surgery, Buddhist Tzu Chi Medical Foundation Taipei Tzu Chi Hospital New Taipei City Taiwan; ^9^ Department of Exercise and Health Promotion, College of Kinesiology and Health Chinese Culture University Taipei Taiwan

**Keywords:** Alzheimer's disease, dementia, gout, meta‐analysis, vascular dementia

## Abstract

**Background and Aim:**

The relationship of gout with the risk of dementia has been a subject of importance in current studies. There is no comprehensive meta‐analysis regarding their association. Hence, our team performed a systematic review and meta‐analysis to examine the relationship of gout with the risk of dementia.

**Methods:**

We searched PubMed, EMBASE, Scopus, and Web of Science beginning at database commencement till 1 December 2022. For systematic review and meta‐analysis, 2 independent reviewers comprised observational researches that assessed the relationship of gout with risk of dementia. This research pursued the Preferred Reporting Items for Systematic reviews and Meta‐Analyses summarizing guidelines to extract and synthesize data. Our team applied the random‐effects model to determine pooled risk ratios (RRs) with 95% confidence intervals (CIs).

**Results:**

Our study covered 5 observational studies. The pooled RR for the risk of overall dementia was 0.80 (95% CI: 0.58–1.10) with no evidence of publication bias. Moreover, the pooled RR demonstrated lesser risk of Alzheimer's disease (AD) at 0.70 (95% CI: 0.62–0.78) and vascular dementia at 0.68 (95% CI: 0.48–0.95) among patients with gout. An inverse association was observed in Asian people, but there was no relationship of gout with dementia among Western people.

**Conclusion:**

In this meta‐analysis of observational studies, gout was not significantly associated with overall dementia risk, although inverse associations were observed for AD and vascular dementia. These findings suggest potential heterogeneity by dementia subtype and population but should be interpreted cautiously given the observational design. Well‐designed prospective studies and randomized trials are needed to clarify causality and underlying mechanisms.

**Protocol Registration:**

International Platform of Registered Systematic Review and Meta‐analysis Protocols INPLASY2025110018; https://doi.org/10.37766/inplasy2025.11.0018.

## 1 Introduction

Dementia, a very usual chronic neurodegenerative disorder and global health problem, affects more than 55 million people [1, 2]. The number of patients with dementia is increasing significantly: it reached 78 million in 2020 and is expected to reach 139 million in 2050 [3, 4]. This huge number of patients has created growing public health concerns with a substantial economic burden; the estimated cost of dementia was 818 billion USD in 2015 [5]. The prevalence of dementia is higher in women than in men. It is also associated with age: the prevalence is about 0.8% in individuals aged over 65 years and 28.5% in individuals aged over 90 years [6]. The etiology of dementia is complex with combined modifiable effects (diabetes, depression, hypertension, obesity, and smoking) [7, 8, 9, 10] and genetic factors [11]. Therefore, we aim to determine the potential modifiable risk factors for dementia.

Recent evidence has shown that gout is conversely related to the risk of dementia development. Both diseases share similar pathogenic pathways [12]. Uric acid (UA) might reduce oxidative stress [13, 14] and deplete beta‐amyloid [15]. However, the relationship of gout with dementia is conflicting in extensive epidemiological studies, ranging from a positive association [16] to an inverse association [17]. Thus, whether gout increases or decreases the risk of dementia remains unclear.

To address these conflicts, our team used systematic review and meta‐analysis to determine the relationship of gout with dementia risk.

## 2 Methods

Our research protocol was registered in the International Platform of Registered Systematic Review and Meta‐analysis Protocols (ID: INPLASY2025110018). Our team executed a systematic review with meta‐analysis according to the Preferred Reporting Items for Systematic reviews and Meta‐Analyses (PRISMA) reporting guidelines [18, 19]. Details of data information collection are shown in Table S1.

### 2.1 Search Strategy

We conducted a comprehensive and systematic literature search of PubMed, EMBASE, Scopus, and Web of Science from database inception through December 1, 2022. The search strategy was developed to maximize sensitivity and minimize the risk of missing relevant studies by combining controlled vocabulary terms (including Medical Subject Headings [MeSH]) with free‐text keywords related to gout and dementia.

A stepwise Boolean approach was applied, in which outcome‐related terms (including dementia, Alzheimer disease, and cognitive impairment) were first combined using the OR operator and subsequently intersected with exposure‐related terms (gout) using the AND operator. Database‐specific indexing ensured that studies using alternative terminology were captured through concept mapping during retrieval. The full PubMed search strategy, including Boolean operators and term combinations, is provided in Table S2.

To further enhance completeness, we performed manual searches of reference lists from all eligible articles, relevant reviews, and prior meta‐analyses. No language restrictions were applied during the search process.

### 2.2 Eligibility Criteria

Eligibility was restricted to observational studies (case–control, cohort, and cross‐sectional) which evaluated the relationship of gout with the risk of any kind of dementia as a primary outcome. We included studies if (i) their outcome of interest was the risk of dementia, (ii) they had been published in English, (iii) they included at least 40 participants and provided clear information regarding selection criteria for gout and dementia, and (iv) they provided effect sizes with 95% confidence intervals (CIs) and adjusted risk factors. Our study excluded abstracts, letters to editors, case reports, short communications, and reviews.

### 2.3 Selection Process

Two authors screened the titles and abstracts of all recruited articles separately. Connected with the previously developed study selection criteria, these 2 authors separately selected relevant researches. If any disagreement arose in the study selection process, it was settled by consultation with the corresponding author. The very 2 authors established data collection forms to extract the required data from selected studies. Both authors examined the extracted information for duplications by checking the first author's first and last name, publication year, and journal name.

### 2.4 Data Extraction

The primary outcome measure was pooled adjusted risk ratios (RRs) with the respective 95% CIs for the relationship of gout with overall dementia risk. We identified adjusted odds ratios (ORs) and hazard ratios (HRs) with 95% CIs and possible confounding factors. We also extracted the name of the first author, study design, publication year, country, data collection period, follow‐up time, percentage of male and female participants, age, selection criteria for gout and dementia patients, and study population.

### 2.5 Assessment for Risk of Bias

Two authors assessed the risk on the basis of each study applying the Newcastle‐Ottawa Scale (NOS) separately [19, 20, 21]. They categorized the selected researches into low, moderate, and high risk of bias based on the number of points they received. Moreover, they determined the heterogeneity among study‐specific RRs using the *Q* statistic, the *I* ^2^ statistic, and *τ* ^2^. We used the Egger's test method to evaluate publication bias.

### 2.6 Statistical Analysis

We conducted the meta‐analysis by using the relevant studies that provided adjusted ORs/HRs of the relationship of gout with overall dementia. We calculated the pooled adjusted RRs with 95% CIs by using the random‐effects method. We drew forest plots to present the overall effect size visually. Moreover, we generated a funnel plot to show publication bias. We performed all analyses by using the meta‐analysis software.

## 3 Results

### 3.1 Study Selection

**FIGURE 1:**
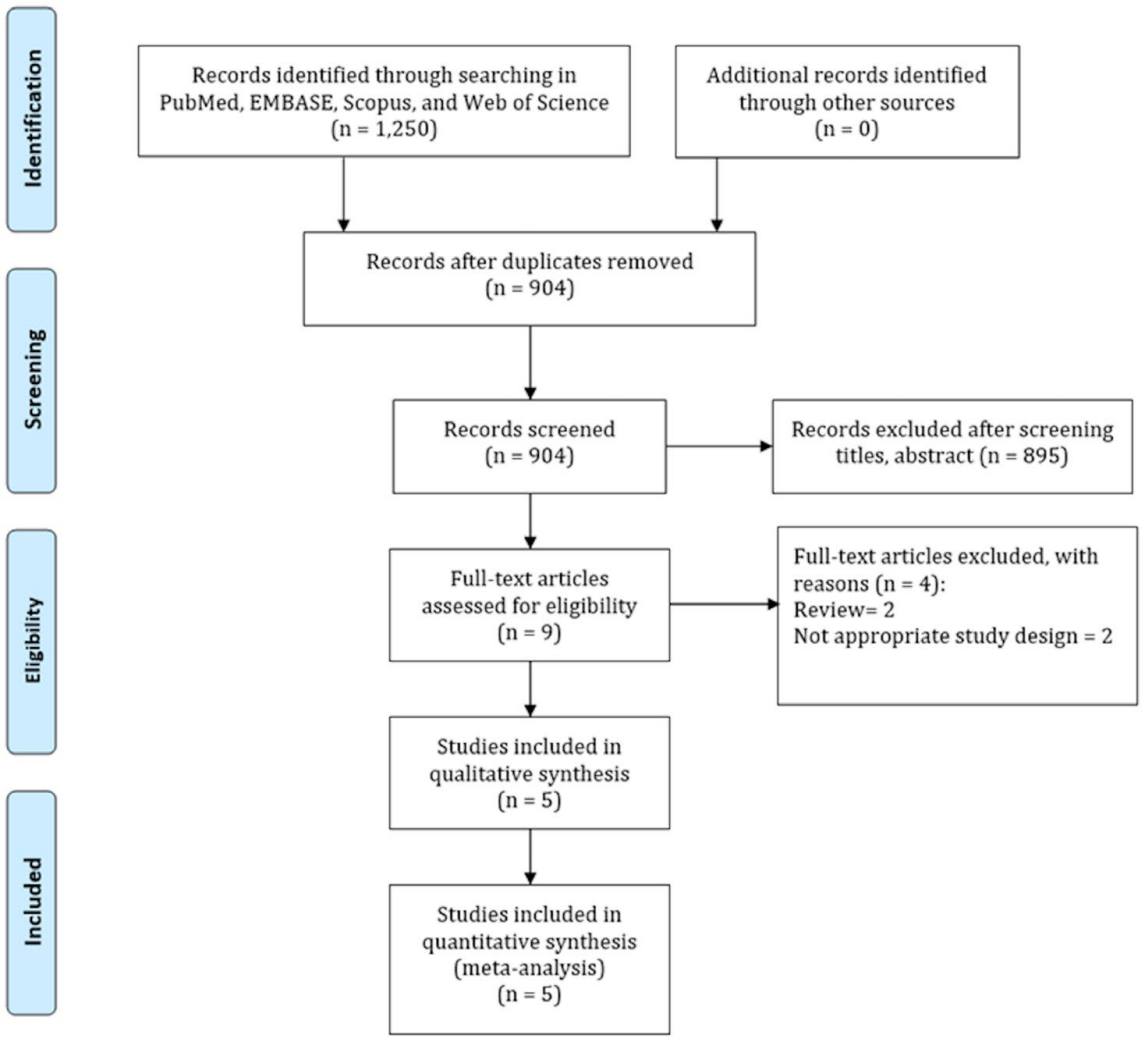
Search strategy for the association between gout and dementia risk.

Initial searches performed in PubMed, EMBASE, Scopus, and Web of Science yielded 1250 studies. After eliminating duplicates and checking titles and abstracts, our team removed 346 articles because of the absence of inclusion criteria. We inspected 9 full‐text articles and checked their citation lists for important articles. From this report, our team eliminated four studies due to review and inappropriate study design. Finally, we included five studies in the meta‐analysis [12, 16, 17, 22, 23]. Figure 1 demonstrates the flow chart of the systematic literature review.

### 3.2 Study Characteristics and Study Quality

We included 5 observational researches in our study (Table 1). The publication year started since 2015 through 2022. Overall, the studies enrolled 227 419 adults with gout who were men and women of all ages. There were five cohort studies [12, 16, 17, 22, 23], which used a standard protocol to select patients with gout and dementia. Two authors determined the quality of the assessed researches by using the NOS, which is suggested by Cochrane to determine non‐randomized studies. The NOS scores ranged from 8 to 9 (9 is the highest score), with a mean of 8.2 and a median and mode of 8.

**TABLE 1:** Observational researches involved in the systematic review and meta‐analysis.

Author	Year	Country	Study design	Study participant (2 groups)	Age (year in 2 groups)	Gender (% male in 2 groups)	Inclusion criteria for gastric cancer	Adjustment	NOS
Kim	2022	South Korea	Cohort	5052/25 260	Reported as group	92.4/92.4	ICD	Age, sex, income, HTN, DM, dyslipidemia, stroke, depression	9
Min	2021	South Korea	Cohort	22 718/113 590	72.29/72.29	62.5/62.5	ICD	Age, sex, CVDs	8
A.Singh	2018	United States	Cohort	111 656/1 601 165	80.0/74.9	32.9/43.3	ICD	Age, sex, HTN, hyperlipidemia, CAD	8
Lu	2016	United Kingdom	Cohort	59 224/238 805	65.3/65.3	70.8/71.1	ICD	BMI, cardiovascular disease, SDI	8
Hong	2015	South Korea	Cohort	28 769/114 742	63.5/63.5	63.4/63.4	ICD	Age, DM, HTN, COPD	8

Abbreviations: BMI, body mass index; CAD, coronary artery disease; COPD, chronic obstructive pulmonary disease; CVDs, cardiovascular diseases; DM, diabetes mellitus; HTN, hypertension; ICD, international classification of diseases; NOS, Newcastle‐Ottawa Scale; SDI, social deprivation index.

### 3.3 Meta‐Analysis

#### 3.3.1 Gout and Dementia Risk

**FIGURE 2:**
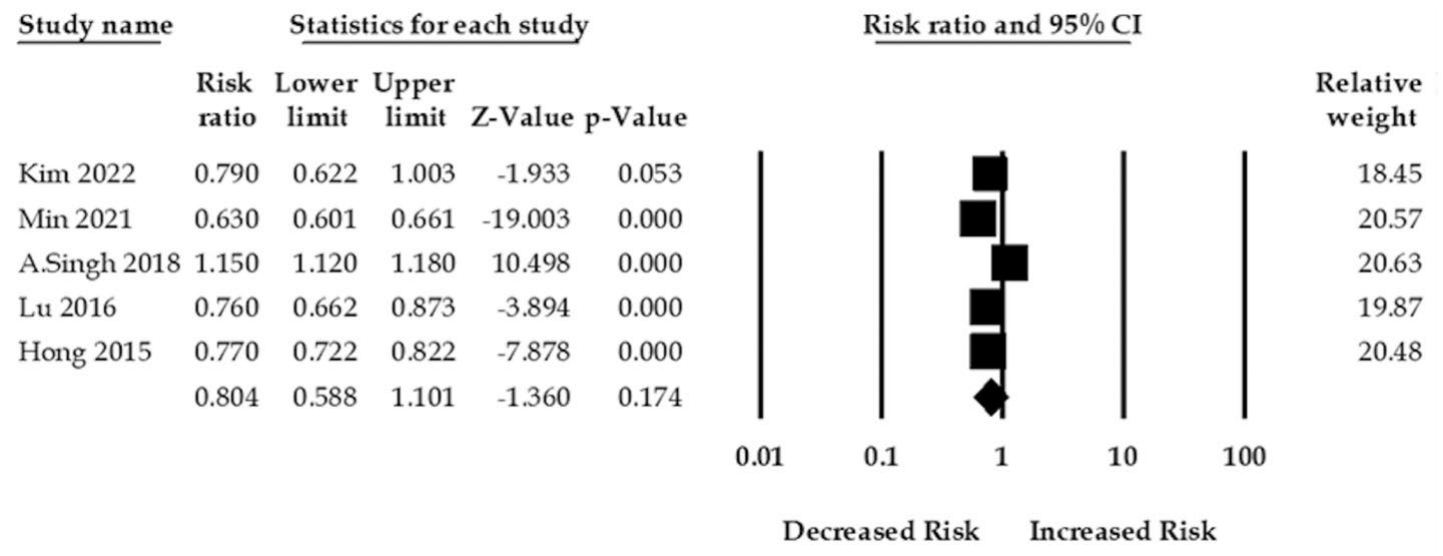
Association between gout and overall dementia risk.

This meta‐analysis comprised five studies with 227 419 gout patients. In the pooled analysis, gout and overall dementia association was not significant with an adjusted pooled RR of 0.80 (95% CI: 0.58–1.10, *p* = 0.17). There was significant heterogeneity within these 5 studies (*Q* = 541.59, *I* ^2^ = 99.26, *τ* ^2^ = 0.12, *p* < 0.001) (Figure 2).

#### 3.3.2 Gout and Alzheimer's Disease Risk

Four studies evaluated the risk of AD in gout patients. There was a negative relationship of gout with AD risk RR of 0.70 (95% CI: 0.62–0.78, *p* < 0.001). There existed moderate heterogeneity in the four studies (*Q* = 7.39, *I* ^2^ = 59.40, *τ* ^2^ = 0.008, *p* = 0.06).

#### 3.3.3 Gout and Cognitive Impairment Risk

Three studies examined the risk of cognitive impairment in patients with gout. The pooled analysis of the overall gout association with cognitive impairment was significant, with a decreased associated risk of 32% for cognitive impairment development RR of 0.68 (95% CI: 0.48–0.95, *p* = 0.02). There existed significant heterogeneity in the three studies (*Q* = 23.08, *I* ^2^ = 91.33, *τ* ^2^ = 0.07, *p* < 0.001).

#### 3.3.4 Subgroup Analyses

We also carried out subgroup analyses depended on research design along with region (Table 2). No stratifications affected the overall effect size and the observed heterogeneity among the included studies. Gout was related with a decreased risk of dementia in the cohort studies. However, the reduction in risk of dementia was insignificant. Gout was related to a diminished risk of dementia in parts of studies accomplished in Asia. Analogous trend was noted in Western population, although this was insignificant neither.

**TABLE 2:** Subgroup analyses for the association between gout and dementia risk.

Studies	Number of studies	Pooled estimates		Test of heterogeneity		
		RR (95% CI)	*p*	*Q*	*p*	*I* ^2^ (%)
All studies	5	0.80 (0.58–1.10)	< 0.001	541.59	< 0.001	99.26
*Study design*						
Cohort	5	0.80 (0.58–1.10)	< 0.001	541.59	< 0.001	99.26
*Region*						
Asian	3	0.71 (0.60–0.84)	< 0.001	25.40	< 0.001	92.12
Western	2	0.94 (0.62–1.41)	0.76	33.35	< 0.001	97.00

### 3.4 Publication Bias

**FIGURE 3:**
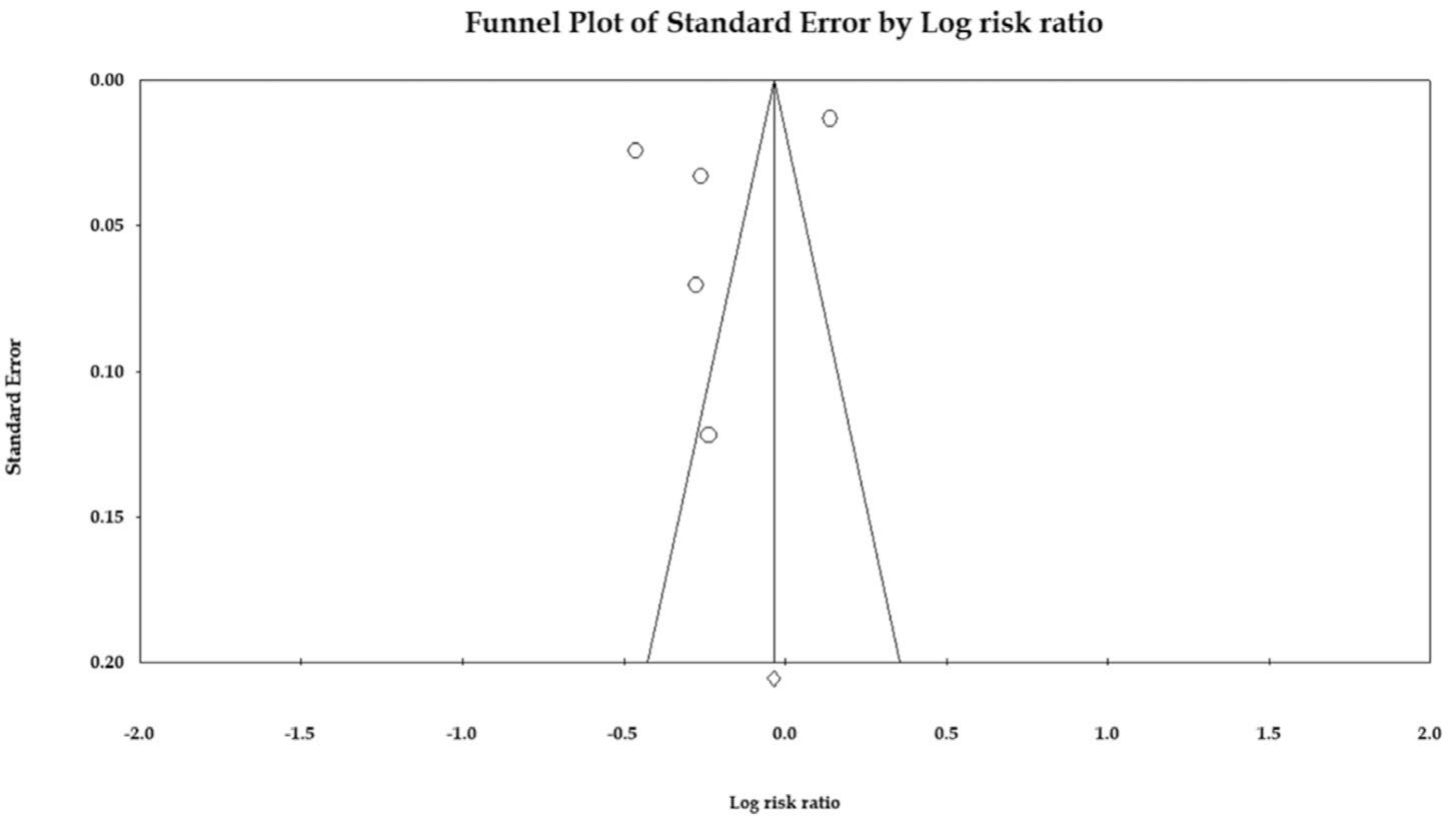
Funnel plot for the association between gout and dementia risk.

Figure 3 shows no publication bias significantly, both quantitative by Egger's regression test (*p* = 0.34) and visual representation (funnel plot).

## 4 Discussion

Our study provides an up‐to‐date evaluation of the relationship of gout with the risk of dementia. We found results from population‐based observational studies evaluating the potential protective effect of gout on the risk of dementia. Moreover, there was an inverse risk of AD and cognitive impairment in patients with gout. The findings from studies carried out in Asian countries indicated an inverse risk of dementia among people with gout. There was no relationship of gout with dementia risk from studies published in Western countries.

The possible biological mechanisms behind the reported inverse relationship of gout with dementia remain unclear. A previous study mentioned that UA might have antioxidant properties [24], namely potential peroxynitrite and hydroxyl radical scavengers to reduce oxidative stress [25]. The neuroprotective effects of gout can be explained by UA that is linked to suppression of oxyradical accumulation and preservation of mitochondrial function [15]. UA likely inhibits the cytotoxic action of lactoperoxidase [26] as well as stops free radical induced deoxyribonucleic acid destruction [27].

A meta‐analysis reported that abnormal oxidative stress in serum, erythrocytes, and circulating lymphocytes is a key factor for AD [28]. UA is related to the elevated accumulation of oxygen and hydroperoxyl radicals, which are able to make iron ions complexes stable [29, 30]. However, the reduced level of UA is linked to weaker protection of oxidative stress, which might lead to an increased risk of cognitive dysfunction. Latourte et al. [31] evaluated the relationship of blood UA with dementia and showed a more powerful relationship with vascular or mixed dementia HR of 3.66 (95% CI: 1.29–10.41) than AD HR of 1.55 (95% CI: 0.92–2.61). Previous studies reported that C‐reactive protein (CRP) is also a biomarker of inflammation and a higher level of CRP in the brain can lead to all‐cause dementia with OR of 3.0 (95% CI: 1.2–7.3) [32]. Moreover, Hsu's research team [33] performed one prospective investigation to examine the relationship of CRP with the risk of dementia in geriatric Asian participants. The investigation showed a 55% higher risk of dementia with HR of 1.55 (95% CI: 1.21–2.00) among patients with a higher CRP level compared to those with a normal CRP level. However, it is reported that gout is related to an elevated level of inflammatory markers including CRP, interleukin (IL)‐1, and IL‐6 [34, 35].

There are some strengths in our study. First, this is a pioneer systematic review and meta‐analysis that has evaluated the relationship of gout with risk of dementia. Second, we found a regional effect regarding the relationship of gout with risk of dementia. Nevertheless, our research has several limitations. First, we included only five articles to assess the risk of dementia among people with gout. Second, we only included observational studies to evaluate the risk of dementia and all the studies were population‐based cohort studies. Therefore, there is a limitation to compare differences between the two study designs (case–control and cohort). Case–control studies are needed to calculate and compare the actual effect size. Third, we could not evaluate the duration of gout and risk of dementia because of data shortage in retrieved articles.

## 5 Conclusion

In this systematic review and meta‐analysis of observational studies, gout was not significantly associated with overall dementia risk, although inverse associations were observed for certain dementia subtypes. The mechanisms underlying these findings remain uncertain and cannot be established from observational data. Further well‐designed prospective studies and randomized clinical trials are needed to clarify causality and to investigate potential biological pathways linking gout and neurodegeneration.

## Author Contributions

Yao‐Chin Wang: conceptualization, methodology, investigation, writing – original draft. Abel Po‐Hao Huang: data curation, resources, validation. Chih‐Wei Huang project administration, visualization. Md. Mohaimenul Islam: software, formal analysis. Woon‐Man Kung: validation, writing – reviewing and editing, supervision. All authors read and approved the final manuscript.

## Funding

The authors have nothing to report.

## Ethics Statement

This article analyzed de‐identified individual participant data from previously published studies. Therefore, formal ethical approval and informed consent are not required. No new data involving human participants or animals were collected by any of the authors. We adhere to all relevant data protection standards and ethical guidelines for secondary data analysis.

## Consent

The authors have nothing to report.

## Conflicts of Interest

The authors declare no conflicts of interest.

## Supporting information


**Table S1:** Details of data information collection from PRISMA 2020 Checklist.

**Table S2:** Search strategy.

## Data Availability

The data that support the findings of this study are available from the corresponding author upon reasonable request.
