# Supplementary material for: Association Between Gout and the Risk of Dementia: A Meta‐Analysis of Observational Studies and Biological Mechanisms
Source: Int J Rheum Dis. 2026 Mar 4;29(3):e70582. doi: 10.1111/1756-185x.70582 (PMC12961178; doi:10.1111/1756-185x.70582)
Supplement: Supplementary file 2 — Table S2: Search strategy. [file APL-29-e70582-s002.doc]

**Supplementary Table S2.** Search strategy.

Database: PubMed

Search period: Database inception to December 1, 2022

Last search date: December 1, 2022

Language restrictions: None

Study design restrictions: Observational studies

| **Step** | **Search terms** | **Records retrieved** |
| --- | --- | --- |
| #1 | *Dementia*[MeSH] OR dementia[tiab] | 259,308 |
| #2 | *Alzheimer Disease*[MeSH] OR “Alzheimer’s disease”[tiab] | 194,391 |
| #3 | *Cognitive Dysfunction*[MeSH] OR “cognitive impairment”[tiab] | 141,108 |
| #4 | *Gout*[MeSH] OR gout[tiab] | 21,752 |
| #5 | #1 OR #2 OR #3 | 402,064 |
| #6 | #4 AND #5 | 159 |

**Search Strategy Description:**

Controlled vocabulary terms (Medical Subject Headings [MeSH]) were combined with free-text keywords searched in (titles and abstracts [tiab]) to capture variations in terminology and indexing. Dementia outcomes were intentionally searched broadly and combined using Boolean “OR” operators before intersection with the exposure term (gout) using the Boolean “AND” operator. This stepwise approach was designed to maximize sensitivity and reduce the likelihood of missing relevant studies.

Equivalent controlled vocabulary (e.g., Emtree terms) and keyword adaptations were applied for EMBASE, Scopus, and Web of Science. Reference lists of eligible articles, relevant reviews, and prior meta-analyses were manually screened to identify additional studies.

# PubMed: 159

# Web of Science: 161

# Scopus: 430

# EMBASE: 559

#Duplicate: 904
